# Enhancing Survival of Mouse Oocytes Following Chemotherapy or Aging by Targeting Bax and Rad51

**DOI:** 10.1371/journal.pone.0009204

**Published:** 2010-02-12

**Authors:** Loro L. Kujjo, Tiina Laine, Ricardo J. G. Pereira, Wataru Kagawa, Hitoshi Kurumizaka, Shigeyuki Yokoyama, Gloria I. Perez

**Affiliations:** 1 Department of Physiology, Michigan State University, East Lansing, Michigan, United States of America; 2 Program of Developmental and Reproductive Biology, Children's Hospital, Helsinki, Finland; 3 Laboratory of Structural Biology, Graduate School of Advanced Science and Engineering, Waseda University, Tokyo, Japan; 4 RIKEN Systems and Structural Biology Center, Yokohama, Japan; Sun Yat-Sen University, China

## Abstract

**Background:**

Therapeutic approaches to preserve fertility in females undergoing cancer treatments are currently ineffective. This is partly due to limited knowledge of the molecular mechanisms that injured germ cells elicit to repair damage and survive or to abort repair and activate biochemical pathways leading to death. So far, we know that following spontaneously occurring or drug-induced DNA damage, the efficiency of DNA repair is a critical determinant of the cell's fate. The protein encoded by the *Rad51* gene is one of several components recruited for homologous recombination-dependent DNA double-strand break repair in both somatic cells and germ cells. Recently, we showed that microinjection of recombinant Rad51 into AKR/J mouse oocytes decreased the extent of spontaneous DNA double-strand breaks, suppressed apoptosis, and restored the developmental competence in AKR/J embryos. Herein we characterized the nature of chemotherapy-induced lesions in oocytes, and the associated individual components of the DNA damage sensor and repair apparatus. For comparison, we also assessed parallel spontaneous changes in aging oocytes.

**Methods:**

Data collected were derived from: analysis of apoptosis; immunodepletion; oocyte microinjections; immunocytochemistry; immunofluorescence; and CHIP-like assays.

**Results:**

Our data show that: (i) DNA damage in oocytes can be induced by both chemotherapy and spontaneously by the aging process; (ii) oocytes possess the machinery and capability for repairing such DNA damage; (iii) Rad51 is a critical player in the repair of both chemotherapy-induced and spontaneously-sustained DNA damage; and (iv) in response to damage, oocytes exhibit an inverse functional relationship between presence of Bax and activity of Rad51.

**Conclusion/Significance:**

Our results establish Rad51 and/or Bax as potential candidates that can be targeted for development of individualized chemotherapeutic interventions that are effective, but minimal in toxicity. The use of Rad51 and Bax modulating compounds could offer women the opportunity to maintain fully functional germ cells despite cancer treatments or aging.

## Introduction

Apoptosis has been implicated as a key mechanism in the depletion of oocytes and follicles (and consequently of ovarian failure) in aged and chemotherapy-treated females [1], [2], [3], [4]. Among specific intracellular events that trigger apoptotic death in oocytes, genomic instabilities such as persistent unrepaired DNA double-strand breaks (DDSB) are thought to be relevant initiators [5], [6], [7]. Nevertheless, several different DNA repair systems have evolved in eukaryotic organisms in order to protect chromosomal integrity [8]. For example, homologous recombination (HR) [9] and non-homologous end joining (NHEJ) have evolved as the most important DNA repair pathways responsible for the repair of DDSB [8]. In this regard, the protein Rad51 performs a vital function in DNA repair by HR [10], and therefore it may play a critical role in oocyte resilience to apoptosis triggered by DNA damage. Indeed, tumor cells overexpressing Rad51 are more resistant to DNA damage induced by chemotherapy [11]. And, we recently reported [7] that microinjection of Rad51 into oocytes of AKR/J mice, a mouse strain deficient in DNA repair, not only reduced the extent of DDSBs, but also suppressed apoptosis; thus strengthening the relationship between the activation of Rad51 and oocyte resistance to cell death.

Bax, a proapoptotic Bcl-2 family member, has also a prominent role in the death of oocytes that sustain DNA damage induced by chemotherapeutic drugs or aging. Inactivation of the *bax* gene noticeably delayed ovarian senescence in female mice [2], [3], and significantly reduced the number of oocytes undergoing spontaneous and chemotherapy-induced apoptosis. Moreover, accumulation of *bax* mRNA transcripts and Bax protein levels has been previously documented in oocytes of aged mice [12].

Thus, we designed the current study to elucidate the extent to which Rad51 and Bax interaction relates to the ability of murine oocytes to repair chemotherapy-induced DNA damage and survive; specific observations include the rate of DNA damage and apoptosis in oocytes cultured with doxorubicin (DXR), and molecular behavior of the DNA damage repair machinery in the presence of Rad51 and Bax proteins.

Improved understanding of oocyte biology is desirable in this new era of personalized medicine, as the quest will lead to biomarker development, innovative therapeutic discoveries, and novel paradigms to deliver individualized therapies that are most likely to be effective but minimal in toxicity.

## Results

### DXR Induces Both Single- and Double-Strand DNA Breaks in Oocytes; Old Oocytes Are More Susceptible to DXR-Induced Apoptosis; and, Some Strains of Mice Are More Prone to DXR-Induced Apoptosis than Others

In previous studies, we assessed the direct effects of the prototypical anticancer drug, doxorubicin (DXR), on mouse oocytes *in vivo* and *in vitro* [6], [13], [14]. Oocytes exposed to DXR rapidly undergo morphological and biochemical changes consistent with the occurrence of apoptosis; and we also provided the first evidence that chemotherapy-induced apoptosis in oocytes is mediated via discrete and specific effector molecules.

**Figure 1:**
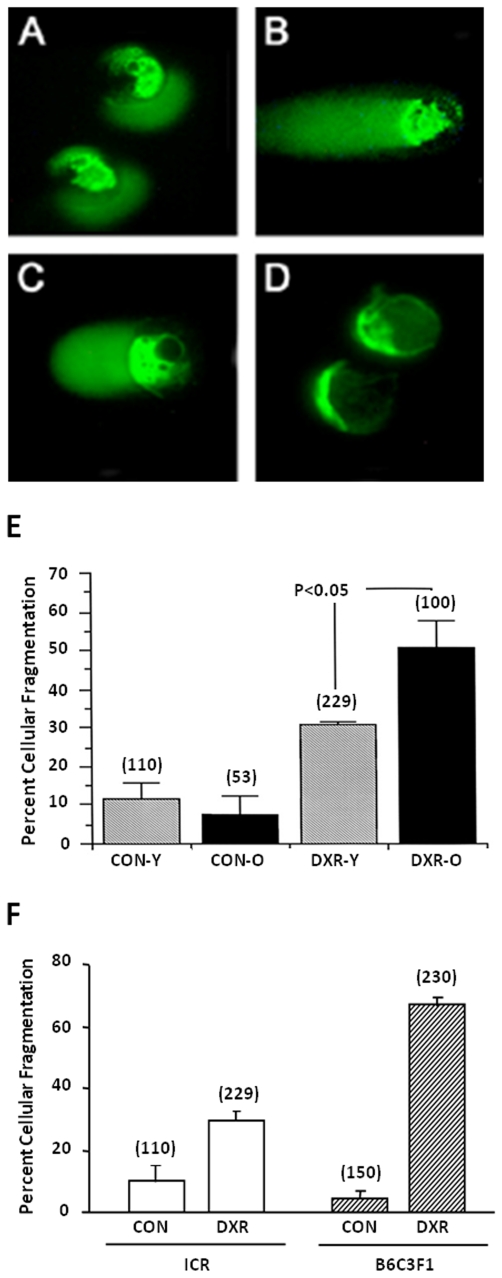
DXR induces both single- and double-strand DNA breaks in oocytes; old oocytes are more susceptible to DXR-induced apoptosis; and, some strains of mice are more prone to DXR-induced apoptosis than others. DNA damage was assessed in oocytes of B6C3F1 mice incubated in the presence of DXR (200 nM) for 24 h. DXR induced both single (**A**) and double-strand DNA breaks (**B**). Addition of 1 mM SAM (sodium aurothiomalate; general nuclease inhibitor) completely prevented double-strand breaks (**D**) but it did not prevent DXR-induced single-strand DNA lesions (**C**). Quantitative assessment of apoptosis in mature oocytes of young (Y; 6 week old) and old ICR mice (O; 43–44 week old), cultured without (control; CON) or with DXR for 24 h, revealed that old oocytes are more susceptible to DXR-induced apoptosis (**E**). And, assessment of apoptosis in oocytes of young ICR and B6C3F1 mice cultured without or with DXR show that oocytes collected from B6C3F1 females are more prone to DXR-induced apoptosis than oocytes collected from ICR females (**F**). After culture, oocytes were fixed and processed for assessment of apoptotic characteristics according to morphological changes (such as cellular condensation, budding, and cellular fragmentation). The percentage of oocytes that showed cellular fragmentation, out of the total number of oocytes cultured in each treatment group, was then determined. The data (mean ± SEM) represent the combined results from at least four independent experiments; the total number of oocytes analyzed per group is indicated over the respective bar.

Herein, we show that DXR induced both single- and double-strand DNA breaks in oocytes of B6C3F1 mice (Figure 1, A–D). We found no apparent differences between the two age groups (young and old) in the degree of DNA damage (data not shown). Nonetheless, the degree of apoptosis induction was different between ages. When oocytes were collected from old (42–44 week old) ICR mice and exposed to DXR, 20% more oocytes died by apoptosis (51±6.6% mean ± SEM) compared with oocytes collected from young (6 week old) ICR mice which sustained 31±0.8% mean ± SEM apoptosis (Figure 1, E). Interestingly, oocytes from various mouse strains responded to DXR with induction of apoptosis although at different degrees (Figure 1, F).

### Oocyte Resistance to DXR-Induced DNA Damage Is Linked to DNA Repair

To uncover the basis of the age- and strain-dependent susceptibility to DXR-induced apoptosis, we assessed oocyte DNA repair. Since irreparable DNA damage triggers apoptosis [7], we reasoned that successful repair of the damage could be linked to increase oocyte resistance to death. We speculated that oocytes collected from mice less susceptible (due to either young age or genetic background) to DXR have the capability to repair a fraction but critical component of the damaged DNA (induced spontaneously or by chemotherapy treatment).

**Figure 2:**
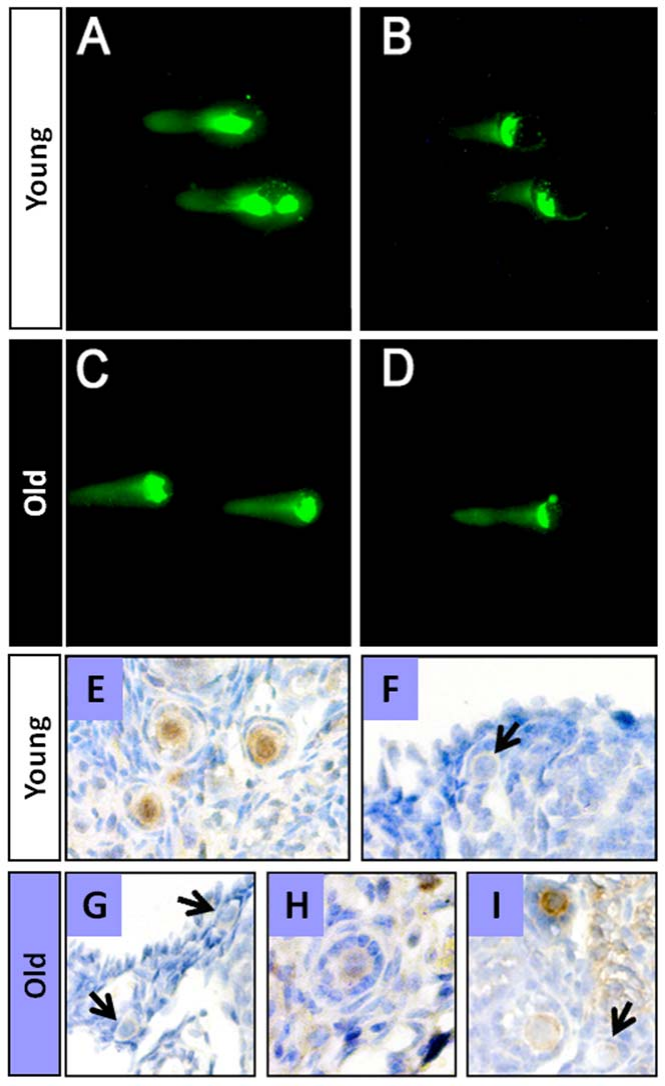
Oocyte resistance to DXR- or aging-induced DNA damage is respectively linked to DNA repair and Rad51. Approximately 80% of oocytes from young and old ICR mice show considerable DNA damage by the time of isolation (respectively, **A & C**). However, after 6 h of incubation oocytes from young mice significantly repaired the DNA damage as evidenced by the decrease in the length of the comets during the same incubation time (**B**; compare A *vs*. B); oocytes from old mice did not show any repair capacity (**D**; compare C *vs*. D). Immunohistochemical localization of Rad51 in ovarian sections from ICR (young and old) mice show that, in young ICR mice (**E, F**), approximately 90% of primordial and primary follicles stained positive for Rad51. While in old ICR mice (**G,H,I**) only 50% or less of the follicles showed a positive reaction. Arrows point to follicles showing a negative reaction.

We consistently observed that a high percentage (80%) of freshly isolated oocytes from young and old ICR mice exhibited substantial DNA damage by the time of isolation (cleaved DNA 52±6.42% mean ± SEM in young, n = 43; compared to 73±7.33% mean ± SEM in old, n = 52; Figure 2A&C). Nonetheless, when these oocytes were cultured *in vitro* for 6 h, oocytes from young mice were able to repair the DNA damage as evidenced by the decrease in the length of the comets (cleaved DNA 8±0.43% mean ± SEM; more than 50% decrease; n = 65; Figure 2B). In contrast, the oocytes from old mice did not show any repair capacity (cleaved DNA 89±5.31% mean ± SEM, n = 41; Figure 2D). The ability of oocytes to repair spontaneously damaged DNA was also seen in the other strains (B6C3F1 and C57BL/6, data not shown).

### Rad51 Is a Critical Player in the Repair of DNA Lesions, Not Only during Spontaneous DNA Damage, but also after Damage Elicited by DXR

Next, we determined whether the capacity to repair DNA damage correlates with expression of DNA repair enzymes (e.g. Rad51) and sensitivity to chemotherapy. Rad51 was chosen based on our earlier studies [7] where we found that this DNA repair protein plays a critical role in oocyte developmental potential and susceptibility to apoptosis in AKR/J mice that are deficient in DNA repair. To this end, we found that resistance to death, by oocytes collected from young ICR and B6C3F1 females was highly correlated with the presence of the DNA repair enzyme, Rad51. Figure 2 (panels E&F) shows that in ovaries from young ICR mice, which are less susceptible (lower death rate) to DXR, at least 90% of the primordial oocytes stained positive for Rad51 (89±2.5% mean ± SEM, Rad51-positive primordial and primary follicles; 81 total number of follicles counted; 9 randomly selected sections/3 mice). However, in ovarian sections of old ICR mice (Figure 2G–I) which are more susceptible (high death rate) to DXR, only 50% or less of primordial follicles stained positive for Rad51 (48±3.2% mean ± SEM; 79 total number of follicles counted; 9 randomly selected sections/3 mice). Similar results were observed in ovaries of other strains susceptible to DXR (e.g. B6C3F1, data not shown).

**Figure 3:**
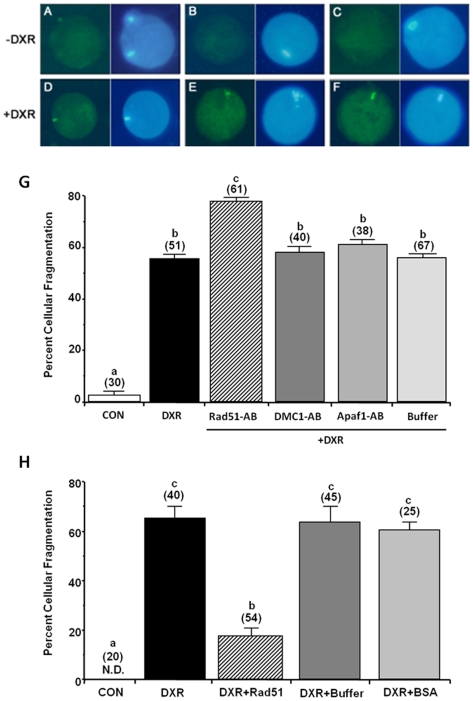
Oocyte resistance to DXR-induced DNA damage is linked to Rad51. Representative photo-micrographs of control (**A–C**; no DXR; n = 38) and DXR-treated (**D–F**; +DXR; n = 45) oocytes are presented. Immunocytochemical localization of Rad51 in mature B6C3F1 oocytes reveal that 15 min after DXR exposure, Rad51 (bright green blots in **D–F**) colocalized with DNA (light blue blots in D–F) in approximately 40% of oocytes. This percentage correlates closely with the number of oocytes that survived chemotherapy *in vitro* (approximately 30–40%). Immunoneutralization of Rad51 rendered 20% more oocytes susceptible to DXR-induced death (**G**), immunoneutralization of DMC1, or Apaf-1, or microinjection of buffer did not affect DXR-induced apoptosis. Conversely microinjection of rRad51 before DXR treatment decreased DXR-induced apoptosis by 70% (**H**), while microinjection of BSA or buffer had no effect on DXR-induced apoptosis. The percentage of oocytes undergoing apoptosis was determined in mature oocytes collected from B6C3F1 mice and cultured without (control; CON) or with 200 nM DXR for 24 h, or after microinjection with Rad51 polyclonal antibody (6 pl of a 200-ng/µl stock per oocyte) followed by DXR treatment (Rad51-AB+DXR), or after microinjection of rRad51 followed by DXR treatment (DXR+Rad51). After culture, oocytes were fixed and viewed microscopically for morphological and biochemical (DNA cleavage) features characteristic of apoptosis. Data shown as mean ± SEM, represent pooled data from at least three independent experiments. The total number of oocytes analyzed per group is indicated over the respective bar. Bars not sharing a common letter are statistically different (*P*<0.05). (N.D. =  none detected).

Furthermore, immunoneutralization of Rad51 in B6C3F1 oocytes, achieved by microinjecting a Rad51 polyclonal antibody (depleted of anti-Dmc1 cross-reactive components), rendered 20% more oocytes susceptible to DXR-induced death as assessed by cellular morphology (Figure 3G); and those oocytes exhibited 30% more DNA damage as well (assessed by comet assay; data not shown). As a negative control we performed immunoneutralization of Dmc1 (polyclonal anti-Dmc1 depleted of anti-Rad51 cross-reactive components by adsorption on Sepharose bound mouse Rad51 protein; [15]) or Apaf-1 before DXR exposure; microinjection of either one of the antibodies (at equivalent concentrations) or buffer did not affect DXR-induced apoptosis (58±2%, n = 40; 60±1.3%, n = 38; 53±2.4%, n = 67; respectively. All data are presented as mean ± SEM).

As expected, *in vitro* treatment of B6C3F1 oocytes with DXR triggered a rapid (within 15 min) accumulation of Rad51 in the DNA (metaphase plate) of mature oocytes (n = 45) (Figure 3, D–F). Remarkably, this early presence of Rad51 correlates highly with the percent of oocytes (approx. 40%) that survive chemotherapy.

Additionally, we examined the role of Rad51 in mouse B6C3F1 oocytes using microinjection of human recombinant Rad51 (rRad51). Microinjection of rRad51 (6 pl of a 3.6-μg/µl stock per oocyte) into oocytes before DXR treatment effectively decreased DXR-induced apoptosis by 70% (Figure 3H). Conversely, microinjection of buffer or BSA did not affect DXR-induced apoptosis (67±3.4% mean ± SEM, n = 45; 60±2.8% mean ± SEM, n = 25; respectively).

From these data we conclude that, the degree of efficiency of DDSB repair is an important factor influencing the chemosensitivity of oocytes, where lack of repair or mis-repair of DSBs most probably leads to apoptosis.

### Inverse Correlation between Efficiency of DNA Repair, Oocyte Survival, and Accumulation of Bax

Given the central importance of Bax in the death of oocytes induced by diverse stimuli [2], [3], [13], [16], we studied oocyte/ovary changes in the availability of Bax and its relationship to DNA damage and repair, under different pathophysiological conditions (e.g. oocytes deficient in DNA repair genetically like in AKR/J females, or due to aging, or due to lack of Bax).

**Figure 4:**
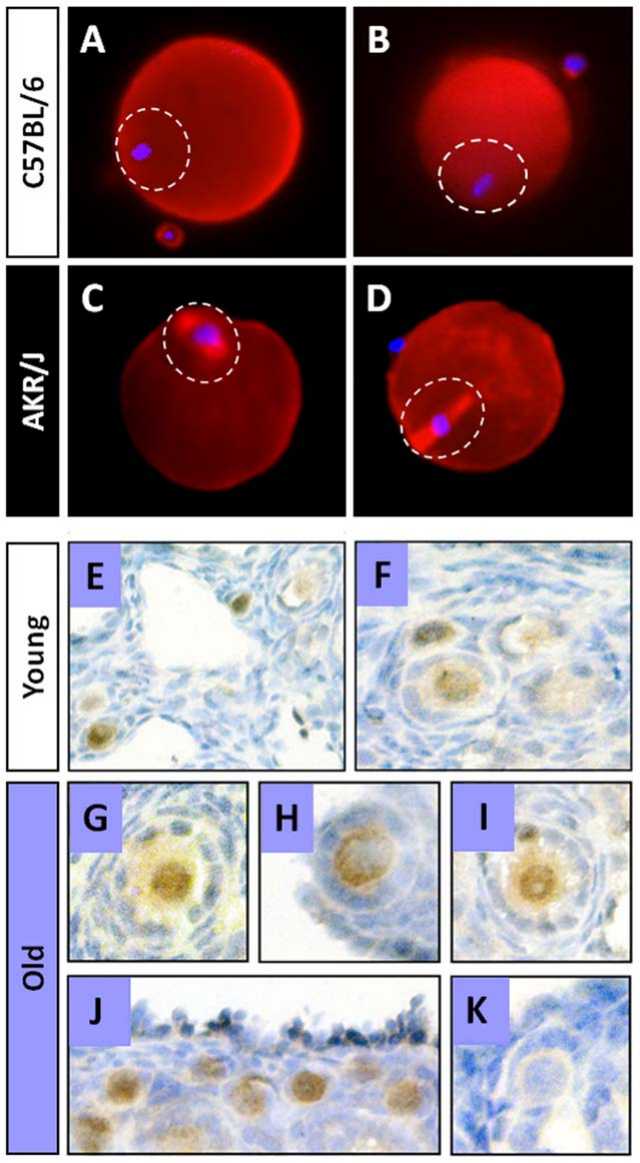
Inverse relationship between the presence of Bax and Rad51. Immunolocalization of Bax in mature (metaphase II) oocytes from young (6 week old) AKR/J (deficient in Rad51-dependent DNA repair) and C57BL/6 mice showed that the levels of Bax protein (red) located nearby the DNA (blue) were lower in oocytes isolated from C57BL/6 (**A&B**), when compared to levels seen in oocytes isolated from AKR/J mice (**C&D**). Moreover, immunohistochemical localization of Rad51 in ovaries of bax-null mice show that the majority of primordial oocytes in both young (**E,F**) and old (**G–J**) mice stain positive for Rad51 (brown color). (**K**) primordial follicle showing negative staining. For comparison, in the wild-type mice Rad51 positive staining decreased with age (Figure 2**G–I**). Mature oocytes were denuded of cumulus cells and immediately (time 0) processed for Bax immunocytochemistry. Oocytes were serially scanned and optical sections were analyzed using Metamorph software (Universal Imaging Corp.). The average pixel intensity for both red and blue channels was calculated by the software for each region. The ratio of red/blue fluorescence (Bax/DNA content) was recorded for each section. Values for each sample were determined as mean ± SEM per oocyte (AKR/J n = 50; C57BL/6 n = 50), and comparison was made between the two strains using Student's t-test. Oocytes (n = 10) devoid of exposure to primary antibody were used as a negative control to determine background noise. (DNA∶blue; Bax∶red or magenta). Immunohistochemical localization of Rad51 was performed in ovaries of bax-null mice young (6 weeks old) and old (42–44 weeks old).

We observed accumulation of Bax protein close to the DNA (at the metaphase plate) of mature oocytes isolated from AKR/J mice that are deficient in DNA repair (Figure 4C&D). The amount of Bax near the DNA of AKR/J oocytes was significantly higher (P<0.05) than in oocytes from C57BL/6 (Figure 4A&B) (ratio Bax/DNA 0.56±0.17 mean ± SEM *vs.* 0.15±0.09 mean ± SEM, respectively; n = 50 oocytes in each group). These experiments were repeated using a primary antibody from Alexis Biochemicals and the results were exactly the same. The ratio of Bax/DNA in AKR/J oocytes was higher (0.63±0.19 mean ± SEM; n = 25) than in oocytes from C57BL/6 mice (0.26±0.02 mean ± SEM; n = 25). Importantly, oocytes isolated from AKR/J mice were unable to repair their spontaneously damaged DNA during a 6-h incubation period. In addition, they accumulated Bax protein close to the DNA, suggesting an inverse relationship between DNA repair and presence of Bax. Therefore, accumulation of unrepairable DNA damage and high levels of Bax protein might be responsible for the high rates of spontaneous apoptosis observed in AKR/J oocytes cultured for 24 h [7].

Oocytes isolated from aged ICR mice are also not able to repair their damaged DNA during a 6-h incubation period (Figure 2D). But, unlike the AKR/J oocytes, no increases in spontaneous apoptotic rates were observed in denuded oocytes from aged ICR mice during the 24 h of culture (see Figure 1E). This suggests that, in the ICR strain the DNA repair mechanisms might not be totally faulty but instead slower or less efficient. Since, we have recently published [6] that a significant increase (5 fold) in bax mRNA transcript and protein occurs in oocytes of aged ICR mice, we hypothesize that Bax is most probably involved in setting the slower rate of repair observed in oocytes of aged ICR mice.

**Figure 5:**
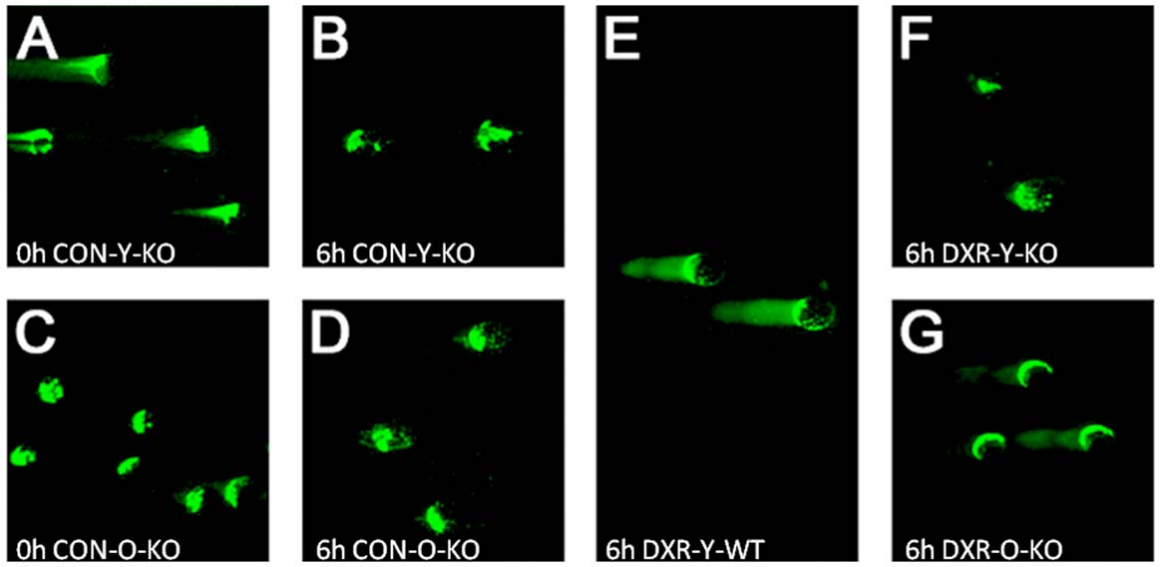
*In vitro* DNA repair capacity of oocytes from *bax*-null mice. Approximately 80% of oocytes from young mutant mice (**A**) showed considerable DNA damage by the time of isolation. However, after 6 h of incubation in control medium these oocytes from young mice (**B**) had significantly repaired the DNA damage as evidenced by the decrease in the length of the comets; and even after 6 h in the presence of DXR their DNA appears still intact (**F**), where as young wild-type oocytes had not after treatment with DXR for 6 h (**E**). The vast majority of oocytes from old bax-null mice showed no DNA damage by the time of isolation (**C**) or after 6 h in culture (**D**); compared with Figure 2 (panels C and D) where oocytes of aged wild-type mice had sustained DNA damage by the time of isolation and were unable to repair it after 6 h of incubation. Oocytes from old bax-null old also sustained a very low level of DNA damage after a 6 h-incubation with DXR (**G**), compared with wild-type oocytes (**E**).

Together, these data suggest an inverse correlation between Bax accumulation and efficiency of DNA repair. It is not surprising therefore, that oocytes from Bax-deficient mice were very efficient in repairing spontaneous and DXR-induced DNA damage *in vitro* (Figure 5), inherently, unlike oocytes from old wild-type mice (ICR shown in Figure 2C&D); or C57BL/6 or B6C3F1 which did not repair their damaged DNA; data not shown.

Approximately 80% of oocytes from young Bax mutant mice showed considerable DNA damage by the time of isolation (time zero; cleaved DNA 35±3.12% mean ± SEM, n = 48; Figure 5A). However, after 6 h of incubation these oocytes had significantly repaired the DNA damage as evidenced by the decrease in the length of the comets (cleaved DNA 3±0.35% mean ± SEM, n = 40; Figure 5B). Interestingly, even after 6 h in the presence of DXR their DNA appears still intact (cleaved DNA 5±0.61% mean ± SEM, n = 46; Figure 5F), compared with DXR-treated wild-type oocytes (cleaved DNA 86±3.46% mean ± SEM, n = 54; Figure 5E). The vast majority of oocytes from bax-null old mice showed no DNA damage by the time of isolation (cleaved DNA 3.5±1.27% mean ± SEM, n = 62; Figure 5C) or after 6 h in culture (cleaved DNA 4.8±2.16% mean ± SEM, n = 58; Figure 5D). In comparison, oocytes of wild-type aged mice had sustained DNA damage by the time of isolation and were unable to repair it after 6 h of incubation (see Figure 2C&D). Oocytes from bax-null old mice also comparatively, sustained a very low level of DNA damage after a 6 h-incubation with DXR (cleaved DNA 29±1.32% mean ± SEM, n = 51; Figure 5G).

In recently published data, we showed that mouse oocytes preserved by disrupting Bax function under normal conditions (i.e., no chemotherapy) are able to dramatically prolong ovarian function into advanced chronological age; in ovaries of aged (32 month old) Bax-deficient female mice follicles ovulate under normal endogenous hormonal conditions. And, the oocytes ovulated are fully capable of fertilization and generation of live offspring [2].

Given these findings, it is reasonable to speculate that the explanation for prolonged ovarian function lies at the molecular level, in that, knocking out the *bax* gene leads to activation or deactivation of a genetic component of the DNA repair mechanism which enables oocytes from the Bax-deficient mice to retain the capability to efficiently repair DNA damage even during aging. The survival of oocytes into advanced chronological age in Bax knockout mice correlates with the presence of the DNA repair enzyme Rad51. This conclusion is supported by the fact that, in wild-type mice the percentage of primordial oocytes staining positive for Rad51 decreases with age, while Bax levels increase with age [12]. Paradoxically, in Bax knockout mice the majority of the primordial oocytes (95–100%) continue to stain positive in both age groups: in young mice, 94±0.6% (mean ± SEM) Rad51 positive primordial and primary follicles from a total number of 85 follicles counted, 9 randomly selected sections/3 mice (Figure 4E&F); in old mice, 96±0.54% (mean ± SEM) Rad51 positive primordial and primary follicles from a total number of 88 follicles counted, 9 randomly selected sections/3 mice (Figure 4G–J). These results suggest that the ability of Bax knockout mice to maintain ovarian activity through advanced ages might be due to the capability of the oocytes to repair DNA damage, and that interaction between Bax and Rad51 appears to play a critical role in the repair mechanism(s). We believe that the efficient DNA repair in the Bax-KO oocytes is not just due to the fact that these oocytes survive longer. Several of our earlier published manuscripts support this point. For example, we have found various ways to make oocytes more resistant to death, either by overexpression of Bcl-2 [17], or by knocking out either caspase-2 [18] or caspase-3 [19], or caspase-12 [20], or by decreasing ceramide levels [12]. However, we haven't found any improvement in DNA repair in any of those oocytes. Only the Bax-KO mice and the Bax-KO oocytes exhibit improved DNA repair, which appears to be mediated by Rad51.

### Bax Interferes with the Binding of Rad51 to Damaged DNA

**Figure 6:**
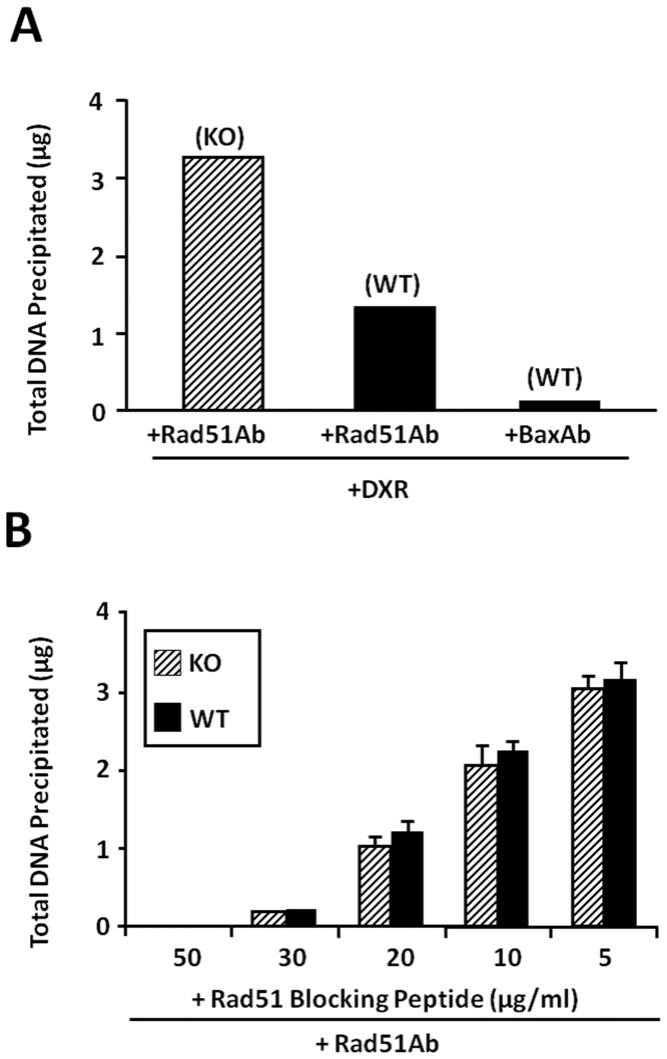
Chromatin immunoprecipitation-like assays in mouse embryonic fibroblasts in the presence or absence of Bax protein. (**A**) MEF-WT and MEF-Bax-KO were treated without or with DXR (200 nM) for 3 h. CHIP assays were adapted to detect the association of the specific proteins (Rad51 or Bax) with DNA. The experiment was performed twice in two different days. (**B**) To exclude the possibility that MEF-WT cells might have less Rad51 protein than MEF-Bax-KO cells, DNA isolated from 10^7^ cells from both (WT and KO) were preincubated with various concentrations of Rad51 blocking peptide before Rad51 immunoprecipitation. No apparent differences between the concentrations of blocking peptide needed to block Rad51 binding in MEF-WT and KO were observed. This indirectly suggests that the amount of Rad51 in these cells is not different from one another. Ab, antibody.

Recently, in an effort to decipher if/and where Rad51/Bax interaction occurs we performed a modification of the chromatin immunoprecipitation (CHIP-like) assays in mouse embryonic fibroblast (MEF) cells; either wildtype (MEF-WT), or lacking Bax (MEF-Bax-KO). The cells were treated without or with DXR (200 nM) for 3 h. CHIP assays were adapted to detect the association of the specific proteins (Rad51 or Bax) with DNA. The highest concentration of immunoprecipitated [2] DNA (3.2 micrograms) was detected in MEF-Bax-KO+DXR following IP with Rad51 (Figure 6A). This fulfills the expectation that in the absence of Bax this protein binds to damaged DNA with high efficiency. Intermediate concentrations of DNA (1.3 micrograms) were precipitated with Rad51 in the MEF-WT treated with DXR; and little or no DNA (0.003 micrograms) was detected in the MEF-WT treated with DXR and IP with Bax suggesting that Bax does not bind to the DNA. Control IPs using preimmune sera exhibited essentially the same results as mock IPs using no antibody. To exclude the possibility that WT cells might have less Rad51 protein, DNA isolated from 10^7^ cells from both MEF-WT and KO were preincubated with various concentrations of Rad51 blocking peptide before Rad51 immunoprecipitation. As seen under Figure 6B there were no apparent differences between the concentrations of blocking peptide needed to block Rad51 binding in MEF-WT or MEF-KO, which indirectly suggests that the amount of Rad51 in these cells is not different from one another.

In summary, results from these preliminary experiments show that under certain molecular conditions, Bax interferes with the binding of Rad51 to damaged DNA. The interaction of Bax/Rad51 seems to occur close to the chromatin (Figure 4C&D), but Bax doesn't appear to bind to DNA (Figure 6A). Although encouraging, these results still need conformation in oocytes.

## Discussion

Understanding the mechanisms underlying oocyte susceptibility to both induced (e.g. chemotherapy) and physiological (age-related) apoptosis can help us to design appropriate interventions to lessen or postpone ovarian follicle depletion and its consequences in women undergoing anticancer treatments or menopause.

Herein, we observed that the apoptotic response of germ cells to DXR differed in scale depending on the mouse strain as well as age of the female. Both phenomena resemble the situation in humans and therefore make the oocyte mouse model a suitable one for studying individual variations to chemotherapy as well as deciphering the role of aging.

Importantly, our findings confirmed that DXR-induced oocyte apoptosis depends on the efficiency of the DNA repair machinery, which in turn declines with age. In oocytes, age-dependent decreases in the efficiency of DNA repair have been linked to changes in gene expression and increases in aneuploidy rates [1], [2], [21], [22], [23], [24], which in some instances lead to apoptotic death [1], [24]. Here we show that chemoresistance in oocytes is dependent on Rad51 due to its central role in homologous DNA repair of DXR-induced DDSBs. These findings are consistent with our previous results in which microinjection of recombinant Rad51 protein improved the defective DNA repair capacity of freshly isolated oocytes from AKR/J mice, decreasing the amount of existent DDSBs and consequently suppressing spontaneous apoptosis [7]. Down-regulation of DNA repair mechanisms has been associated with susceptibility of tumors to a variety of chemotherapeutic agents [11], [25], [26], [27]. Therefore, up-regulation of Rad51 in oocytes is expected to serve as protection from chemo- and radio-therapy by promoting repair of DDSBs.

However, we cannot discard the fact that higher susceptibility of oocytes from old mice to DXR exposure may also derive from other age-related differences (e.g. in oxidative stress resistance or free-radical detoxification pathways [21]) since DXR induces free radical formation, lipid peroxidation and, direct membrane effects as well [6].

An interesting fact we uncovered is that, an inverse functional relationship between presence of Bax and ability of Rad51 to reach damaged DNA is inherent in oocytes. This suggests that the lower rate of DXR-induced and spontaneous DNA damage observed in oocytes from Bax-deficient mice (both young and old) is possibly due to the maintenance of Rad51 activity (and consequently more efficient DNA repair). Downregulation of HR by Bax and Bid has been already reported by Dumay et al. [28] who found that this control occurs independently of Rad51 degradation. But, no attempts were made to determine if Bax perhaps affects transcription, translation, nuclear transport or foci formation of Rad51. What is known though, is that following DNA damage, Bid is phosphorylated by the DNA damage kinase ATM (Ataxia telangiectasia mutated protein kinase). This activates a checkpoint control, which signals the cell to undergo cell cycle arrest and initiate DNA repair.

Therefore, considering the aforementioned discussion, we propose that following DNA damage, and in the absence of Bax, a still unknown molecule activates ATM, leading to a cascade of reactions that culminate in cAbl tyrosine kinase-mediated phosphorylation of Rad51, and consequently higher rates of DNA homologous repair. The evidence that DDSBs are not efficiently detected in older mice because of a lower activity of the ATM kinase supports this idea [29]. Moreover, previous studies have demonstrated that ATM is required for the Rad51 foci assembly induced by ionizing radiation [30] and that phosphorylation of Rad51 by cAbl tyrosine kinase (necessary for its interaction with Rad52) is probably through the formation of a complex with ATM [31], [32].

An alternative hypothesis, based on results from the CHIP-like assays suggests some kind of interaction between Bax and Rad51. In the presence of Bax the binding of Rad51 to damaged DNA is distorted. How does it happen? Is it perhaps by allosteric inhibition? We don't know. But, our data [16], [33] argue for the existence of a still unknown Rad51/Bax-dependent pathway of apoptosis that could be directly controlled from the nucleus in female germ cells.

Our results collectively support a critical role for both Rad51 and Bax in oocyte death both following DXR treatment and during aging. It is thus possible that with a better understanding of the molecular biology of the oocyte, we can develop strategies for manipulating Rad51 and/or Bax as potential candidates for targeting individualized chemotherapeutic interventions that are both effective and minimal in toxicity.

## Materials and Methods

### Animals

Female mice used in all experiments were purchased from Charles River, Taconic, and Harlan Laboratories. Mice were kept in well-controlled animal housing facilities and had free access to water and food. All experiments involving animals described herein were reviewed and approved by the institutional animal care and use committee of Michigan State University.

All the experiments described in this study were intentionally performed using different strains of mice so as to exploit and compare the unique genetics and phenotypes of their ovaries and oocytes with the ultimate aim of reconciling one model for mechanism(s) of DNA repair and its relation to oocyte loss. This strategy allowed us to study individual variations in responses to chemotherapy, and to dissect the effects of genetic background under specific experimental conditions.

Oocytes collected from different strains of mice show diverse phenotypic characteristics, briefly: (1) B6C3F1 (young and old) mice: oocytes from these mice exhibit very low rates of spontaneous apoptosis, and the quality of their DNA is very good at the time of isolation. This strain is ideal for induction of oocyte DNA damage and apoptosis. (2) ICR (young and old) mice: compared with B6C3F1 and C57BL/6, oocytes from ICR mice undergo higher rates of spontaneous apoptosis in culture. In addition, their oocytes display higher rates of DNA damage by the time of isolation. Nonetheless, oocytes from young ICR mice (as also happens in the other mouse strains except AKR/J) are able to repair the initial DNA damage during a 6 h incubation period, but, oocytes from aged mice are not. This mouse strain is therefore used for most of the aging and spontaneous DNA repair studies. (3) AKR/J (young) mice: at the time of isolation, oocytes collected from these mice already show higher rates of DNA damage, in addition to higher rates of spontaneous apoptosis. The DNA damage is not repaired during incubation time. This strain of mice is therefore ideal for studying the role of DNA repair enzymes and Bax in the spontaneous induction of apoptosis. (4) C57BL/6 (young and old) mice: this is the genetic background of the bax KO mice.

### Oocyte Collection and Culture

Female mice were superovulated with 10 IU of equine chorionic gonadotropin (eCG; Professional Compounding Centers of America, Houston, TX) followed by 10 IU of human chorionic gonadotropin (hCG; Serono Laboratories, Norwell, MA) 46 h later. Mature oocytes were collected from the oviducts 16 h after hCG injection. Cumulus cell-enclosed oocytes were denuded of cumulus cells by a 1-min incubation in 80 IU/ml of hyaluronidase (Sigma, St. Louis, MO), followed by three washes with culture medium.

All oocyte cultures were carried out in human tubal fluid (HTF; Irvine Scientific, Santa Ana, CA) supplemented with 0.5% bovine serum albumin (BSA, fraction V; Gibco-BRL Life Technologies, Grand Island, NY). Oocytes were cultured in 0.1 ml drops of culture medium (10 oocytes/drop) under paraffin oil (Specialty Media, Phillipsburg, NJ), and incubated for up to 24 h at 37 C in a humidified atmosphere of 5% CO2–95% air. Assignment to treatment groups was carried out at random, and incubations performed without or with freshly made DXR (200 nM; Alexis Biochemicals, San Diego, CA). Oocyte morphology was evaluated 24 hours later.

### Analysis of Apoptosis

At the end of the culture period, the oocytes were fixed and evaluated as detailed previously [6], [13], [14], for characteristics of apoptosis, mainly morphological changes (*e.g*., condensation, budding and cellular fragmentation) and biochemical alterations (*i.e*., DNA cleavage; Comet Assay Kit, Trevigen, Gaithersburg, MD). The percentage of oocytes that underwent apoptosis out of the total number of oocytes cultured per drop in each experiment was then calculated.

### Comet Assay

Pools of mature (metaphase II) oocytes harvested from superovulated young (6 week old) or old (42–44 week old) ICR mice were denuded of cumulus cells and then cultured for 6 h in control medium. DNA damage was assessed by both the alkali and neutral version of the comet assay to detect single and double-strand breaks respectively, according to the manufacturer's instructions (Trevigen, Inc. Gaithersburg, MD). Analysis of the individual comets to generate quantitative data was performed using a computer based program ([34], VisComet; Impuls Computergestutzte Bildanalyse GmbH, Gilching, Germany). The program automatically calculates all measurement parameters including tail moment, tail percent intensity, and tail length; these are widely regarded as the most informative measures of DNA damage. In this paper we report tail percent intensity as percentage of damaged DNA per cell.

### Oocyte Microinjection

Immediately after isolation, denuded oocytes were microinjected with buffer or with Rad51 polyclonal antibody (6 pl of a 200-ng/µl stock per oocyte), or with anti-Dmc1 polyclonal antibody (6 pl of a 200-ng/µl stock per oocyte) depleted of anti-Rad51 cross-reactive components (by adsorption onto Sepharose bound mouse Rad51 protein [15]), or with rRad51 (6 pl of a 3.6-μg/µl stock per oocyte; this protein was purified and tested by Dr. Kurumizaka at RIKEN institute, Japan), or with BSA (at equivalent concentrations). Oocytes that did not survive the microinjection procedure (usually <15%) were discarded. The remaining oocytes were cultured for up to 24 h, followed by assessment of apoptosis as described earlier.

### Immunodetection of Rad51 in Ovaries and Oocytes

Paraffin-embedded ovarian sections (4 µm) from young (6 weeks old) and old (42–44 weeks old) mice were analyzed by immunohistochemistry with high-temperature antigen unmasking. After peroxidase quenching and microwave treatment, sections were incubated with a 1∶50 dilution of Rad51 goat polyclonal antibody (Santa Cruz, sc-6862) overnight at 4 C. Since Rad51 shares a high degree of identity with Dmc1 (disrupted meiotic cDNA; 100), the primary antibody was immunodepleted of components recognizing Dmc1 epitopes by adsorption onto a Dmc1 bound Ni-NTA agarose column; this treatment abolishes the cross reactivity with the Dmc1 [15]. The specificity of the antibody was assessed in histological sections where the first antibody was omitted from the reaction (negative control). Sections were washed and then incubated with a 1∶200 dilution of biotinylated donkey anti-goat antibody for 45 min at room temperature (RT). Next, the sections were sequentially washed and incubated for 45 min at RT with horseradish peroxidase-conjugated streptavidin, washed and reacted with ice-cold 3,3′ diaminobenzidine (0.5 mg/ml with 0.03% hydrogen peroxide) at RT. Sections were then counter-stained with hematoxylin and analyzed by light microscopy.

Mature oocytes were isolated and cultured without (control; CON) or with 200 nM DXR. During the first hour of incubation oocytes were fixed every five minutes and processed for detection of DNA (DAPI staining) and co-localization of Rad51 by immunocytochemistry as described, with the modification that the secondary antibody was FITC-labeled. Therefore the signal was visualized by fluorescence microscopy. No signal was detected when the first antibody was omitted from the reaction (negative control).

### Immunodetection of Bax in Oocytes

The procedures were the same as described for Rad51, with the modification that the secondary antibody was conjugated with Alexa 594 or Alexa 546 (Invitrogen). Two different primary antibodies were tested, Bax N-20 rabbit polyclonal (Santa Cruz, sc-493) and Bax mouse monoclonal (Alexis Biochemicals, ALX-804-424). Imaging was performed by a spinning-disk confocal system mounted on a Nikon TE-2000 microscope at 40× (numerical aperture (NA) 1.3) and 100× (NA 1.3) magnifications. Oocytes were serially scanned and optical sections were analyzed using Metamorph software (Universal Imaging Corp.) and ratio of red/blue fluorescence (Bax/DNA content) was recorded for each section. Also, two different cytoplasmic areas were delineated to use as background fluorescence. The different regions were then transferred to the red and blue channels, and the average pixel intensity calculated by the software for each region. For analysis, each region's fluorescence intensity was divided by the average of the two cytoplasmic regions. Oocytes from the two mouse strains C57BL/6 and AKR were stained at the same time and imaged during the same day. Staining and imaging was repeated with two different batches of oocytes for a total of 75 oocytes stained for each strain (50 oocytes/Sta. Cruz antibody and 25 oocytes/Alexis antibody).

### Chromatin Immunoprecipitation Assays (CHIP-Like Assays)

Mouse embryonic fibroblasts (MEF) derived from both wildtype (MEF-WT), and Bax knockout (MEF-Bax-KO) mice were treated without or with DXR (200 nM) for 3 h. CHIP assays were adapted to detect the association of the specific proteins (Rad51 or Bax) with DNA. MEF were fixed with formaldehyde (to cross-link protein-DNA complexes) at 3 h after DXR exposure, when DXR-induced damage is robust and activation/mobilization of Rad51 and Bax are also probably still occurring. Sonicated nuclear lysates were immunoprecipitated with antisera directed against Rad51 or Bax (at concentrations ranging from 10 to 50 µg/ml at 4°C overnight), or with protein G-agarose beads alone (no antibody). Protein-DNA complexes were eluted; cross-links were reversed by adding NaCl (200 mM) and 10 µg of RNase A and incubating at 65°C overnight. After ethanol precipitation, samples were digested with proteinase K (Boehringer) at 42°C for 2 h and then extracted with phenol-chloroform. A second ethanol precipitation was performed, after which the DNA samples were resuspended in 20 µl of TE, and DNA concentrations were determined. The experiment was performed twice in two different days.

### Statistical Analysis

All experiments were independently replicated at least three times (unless stated otherwise) with different sets of mice. Data not differing significantly were pooled by groups. The combined data from the replicate experiments were subjected to a one-way analysis of variance followed by Scheffe's F-test, Fisher's exact test or Student's *t*-test. Differences between group means were considered statistically significant at *P*<0.05 (*) or at *P*<0.001 (**). The data depicted in graphs represent the mean ± SEM of the combined data.
